# Tumor-specific T cell-mediated upregulation of PD-L1 in myelodysplastic syndrome cells does not affect T-cell killing

**DOI:** 10.3389/fonc.2022.915629

**Published:** 2022-08-05

**Authors:** Valentina Ferrari, Alison Tarke, Hannah Fields, Tiffany N. Tanaka, Stephen Searles, Maurizio Zanetti

**Affiliations:** ^1^ PersImmune, Inc., San Diego, CA, United States; ^2^ Moores Cancer Center, Department of Hematology and Oncology, University of California San Diego, La Jolla, CA, United States; ^3^ The Laboratory of Immunology, Department of Medicine and Moores Cancer Center, University of California San Diego, La Jolla, CA, United States

**Keywords:** myelodyslastic syndromes, neoantigens (neoAgs), adoptive cell transfer (ACT), immune check inhibitor (ICI), unfolded protein response (UPR)

## Abstract

The PD-1:PD-L1 axis is a binary interaction that delivers inhibitory signals to T cells, impeding both immune surveillance and response to immunotherapy. Here we analyzed a phenomenon whereby tumor-specific T cells induce PD-L1 upregulation in autologous MDS cells in short-term culture, through a mechanism that is cell-contact-independent and partially IFNγ-dependent. After investigating a panel of small-molecule inhibitors, we determined that PD-L1 upregulation was attributed to the PKR-like ER kinase (PERK) branch of the unfolded protein response. Interestingly, we found that the cytotoxic capacity of tumor-specific T cells was not impaired by the expression of PD-L1 on MDS target cells. These results highlight a little appreciated aspect of PD-1:PD-L1 regulation in hematologic cancers and indicate that this phenomenon, while likely to hinder autochthonous immune surveillance, may not be an obstacle to immunotherapies such as personalized adoptive T-cell therapy.

## Introduction

Myelodysplastic syndromes (MDS) are a heterogeneous group of hematologic malignancies characterized by bone marrow failure and peripheral cytopenia. The 2-year survival rate is 15% for patients with higher-risk MDS who have failed standard-of-care therapy with hypomethylating agents (HMAs), and the median survival rate is less than 6 months for higher-risk patients who are refractory to, or have relapsed on HMA treatment (1). Currently, the only curative treatment for these patients is hematopoietic stem cell transplant (HSCT). However, due to older age and comorbidities, patients with MDS often are not eligible for HSCT (2, 3). More effective, less toxic therapies are sorely needed for elderly patients affected by MDS, and therapies that harness the patient’s endogenous immunity and avoid non-specific cellular damage have increasing clinical success in a variety of malignancies.

Personalized therapy by adoptive cell transfer (ACT) of autologous T cells specific for MDS tumor cell neoantigens is an attractive new approach to treat MDS patients (4, 5). A potential drawback to this approach is the induction of PD-1 on the surface of tumor-specific T cells and the presence of PD-L1 on tumor targets, resulting in inhibitory interactions that suppress T-cell responses. PD-L1 is a surface antigen that plays a major role in suppression of the adaptive immune system by binding to its ligand PD-1, which is present on the surface of activated T cells. While the PD-1:PD-L1 interaction is an essential mechanism that helps prevent autoimmunity, reports have shown that cancer cells can also upregulate PD-L1 on their surface. Thus, cancer cell upregulation of PD-L1 allows them to effectively evade immune surveillance by ligating the PD-1 receptor on potentially tumor-specific T cells. The regulation of PD-L1 expression is a topic of significant interest owing to the success of immune checkpoint inhibitor (ICI) therapy in solid tumors such as melanoma and non-small cell lung carcinoma (6). Ongoing clinical trials evaluating the efficacy of ICI among MDS patients are based on data suggesting that these molecules are expressed on tumor and immune cells after HMA treatment (7). However, the relationship between tumor-expressed PD-L1 and sensitivity to ICI therapy is not yet understood, warranting an in-depth analysis to better inform future combination treatment regimens (8).

Here we analyzed potential mechanisms for PD-L1 upregulation on MDS tumor cells in the presence of autologous tumor-specific T cells to better understand if ICI therapy can be applied as a combination strategy with ACT. We found that in MDS tumor cells the upregulation of PD-L1 is contributed by both the unfolded protein response (UPR) and to a lesser extent by IFNγ. We also found that both PD-L1-expressing and PDL-1-negative MDS tumor cells were killed by T cells with equal efficiency. These results have implications for immune-based treatment strategies for patients with MDS.

## Materials and methods

### Patient samples

Patients (n = 8) with intermediate-, high-, and very high-risk MDS were recruited at the UCSD Moores Cancer Center (NCT03072498), and samples were collected under a protocol approved by the UCSD Institutional Review Board (IRB) and in accordance with the Declaration of Helsinki. Prior to initiating standard-of-care therapy, a leukapheresis for mononuclear cell collection was performed using a COBE Spectra or Optia system.

### Induction of tumor-antigen-specific T cells

Tumor-antigen (TA)-specific T cells were generated as previously described (4). Briefly, CD8^+^ and CD4^+^ T cells were stimulated with autologous monocyte-derived dendritic cells (moDCs) pulsed for 2–4 h at 37°C with either irradiated, autologous MDS cells or (1–5 μM) of neoantigen peptide pools. Cells were fed every 3–4 days with 50–100 U/ml IL-2. The T cells were restimulated once prior to testing in T-cell killing (TCK) assays.

### TCK assay

Assay was performed as previously described (4). Briefly, target MDS cells labeled with CellTrace Violet fluorescent dye (Life Technologies, Carlsbad, CA) were combined with TA-specific T cells and incubated at 37°C. After 16–22 h, cells were stained with 7-AAD to discriminate live and dead cells. The assay was analyzed on an iQue Screener PLUS (Intellicyt), and the percentage of target cells killed was calculated as ([live cells in control − live cells in test samples]/live cells in control) × 100 = cytotoxicity (%).

### IFNγ release

Supernatants were collected after a 16- to 18-h coculture of TA-specific T cells with their autologous MDS cells. IFNγ was captured using the QBeads assay (IntelliCyt) following the manufacturer’s instructions and quantified on an iQue Screener PLUS (IntelliCyt).

### Transwell culture system

MDS cells were plated alone on the bottom portion of the well prior to adding a Transwell insert. Tumor-specific T cells and MDS cells were cocultured at a 10:1 effector:target ratio on the top portion of the Transwell insert. After 24 h, the MDS cells from the bottom portion of the Transwell were removed and PD-L1 was assessed by flow cytometry.

### Inhibition with small molecules

MDS cells were pre-incubated at 37°C for 4 h with small molecules 4µ8C (30 nM), ISRIB (1 µM), and Salubrinal (70 µM) prior to coculture with autologous tumor-specific T cells, without the removal of the small molecules. After 18–20 h of coculture, cells from each condition were harvested and stained with CD3, CD4, CD8, PD-L1, CD33, and 7AAD, according to manufacturer’s instructions. Samples were acquired on an iQue Screener PLUS and analyzed using ForeCyt software (IntelliCyt).

## Results

### Patient-derived MDS cells upregulate PD-L1 in the presence of autologous TA-specific T cells in a cell-contact-dependent manner

**Figure 1:**
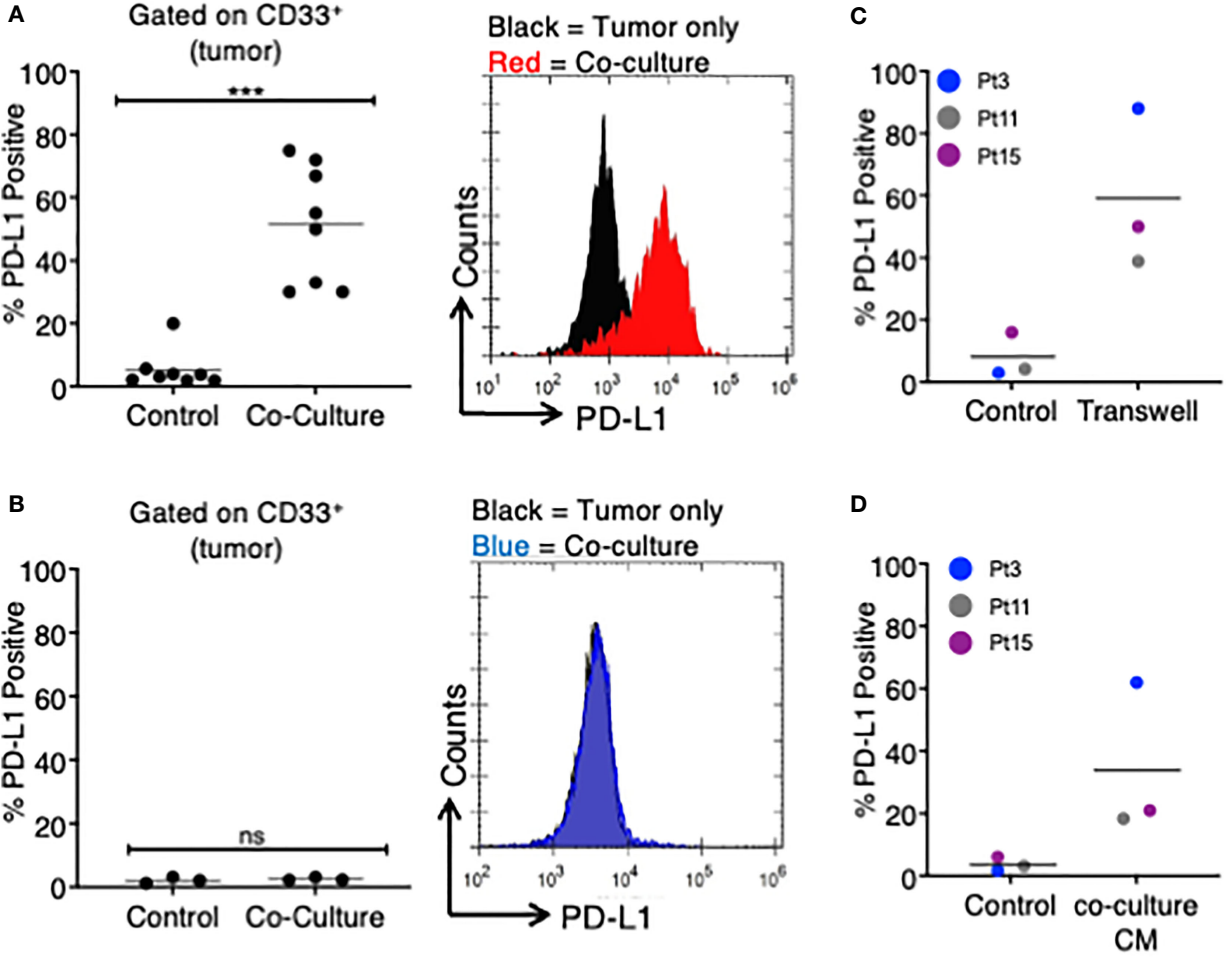
MDS cells upregulate PD-L1 in the presence of autologous TA-specific T cells. MDS cells from eight patients were analyzed for PD-L1 expression by flow cytometry in various experiments. **(A)** PD-L1 was measured at baseline (control) or after an 18–20-h coculture with autologous tumor-specific T cells (n = 8). A representative histogram plot is shown to the right. **(B)** A subset of three patients was analyzed for PD-L1 expression at baseline (control) or after an 18–20-h coculture with T cells activated with viral antigens (CEF: CMV, EBV, and flu peptides; coculture). A representative histogram plot is shown to the right. **(C)** MDS cells from three patients were cultured in a Transwell system (with MDS cells in the lower chamber and MDS:T-cell coculture in the upper chamber), and PD-L1 expression is shown. **(D)** MDS cells from three patients were analyzed for PD-L1 expression after 18–20 h of treatment with coculture conditioned medium (CM) from their respective MDS/autologous tumor-specific T-cell coculture. ***p < 0.001 by paired t test. ns, not significant.

We studied eight patients with MDS, four of which were subject to whole-exome and transcriptome sequencing (4). To investigate MDS:T-cell interactions, we first induced tumor-antigen (TA)-specific T cells utilizing either irradiated autologous MDS cells or neoantigen-derived peptides (4) (**Table 1** **)**. After coculturing TA-specific T cells with autologous MDS cells, we found that an average of 51.1% of MDS cells were PD-L1 positive (range 30%–75%), while only 5.3% of MDS cells expressed PD-L1 at baseline (range 1.9%–20%), corresponding to an average 9.6-fold increase in PD-L1 expression (**Figure 1A**).

**Table 1:** Characteristics of immunogenic stimulus and neoantigens (4).

Patient	# of mutations	Immunogenic stimulus	Source gene	Neoantigen
**1**	N/D	XR tumor cells	N/A	N/A
**2**	6	Neoantigen	WDR6	YYNRVHILGEPRPH**F**FGQMFVRLQLLRAV
**3**	8	Neoantigen	CES2	EPTMRLHRLRARLS**V**VACGLLLLLVRGQG
**4**	7	Neoantigen	MIB2	CGPSSRLMGWKPSE**S**RGQSQSFQASGLQP
**7**	N/D	XR tumor cells	N/A	N/A
**9**	9	Neoantigen	HERC2	LGHGNRSPCDRPHVIESLRGIEVVD
**11**	N/D	XR tumor cells	N/A	N/A
**15**	N/D	XR tumor cells	N/A	N/A

XR, irradiated; N/D, not determined; N/A, not applicable.

To determine if PD-L1 would be upregulated by non-TA-specific T cells, we selected three of the eight original MDS patients (Pts 3, 11, 15) and stimulated their T cells with a pool of viral peptides (CEF: cytomegalovirus, Epstein Barr virus, and influenza virus) prior to coculture with autologous MDS cells. In all three patients, the MDS cells did not upregulate PD-L1 in the presence of autologous CEF-specific T cells (**Figure 1B**). This suggests that PD-L1 upregulation is dependent on the presence of TA-specific autologous T cells.

Next, we sought to determine if the upregulation of PD-L1 on MDS cells cocultured with TA-specific T cells was cell-contact dependent. To this end, we cocultured MDS cells in a Transwell system so that tumor cells and T cells were separated by a membrane. In this assay system PD-L1 expression on MDS tumor cells was induced in all three patients analyzed (**Figure 1C**). The magnitude of PD-L1 induction was similar to what was measured in the direct coculture experiments (~7.5-fold increase). Based on this, we transferred the conditioned media (CM) from cocultures of MDS cells with their autologous tumor-specific T cells (referred to as “coculture CM”) onto MDS cells. We found that coculture CM was able to upregulate PD-L1 on MDS cells with an average increase of 9.4-fold in three different patient-derived MDS cells (**Figure 1D**). Together, these data suggest that PD-L1 upregulation is induced by the secretome of MDS:T-cell cocultures.

### Blockade of IFNγ and the PERK branch of the UPR pathway attenuates PD-L1 upregulation on MDS cells

**Figure 2:**
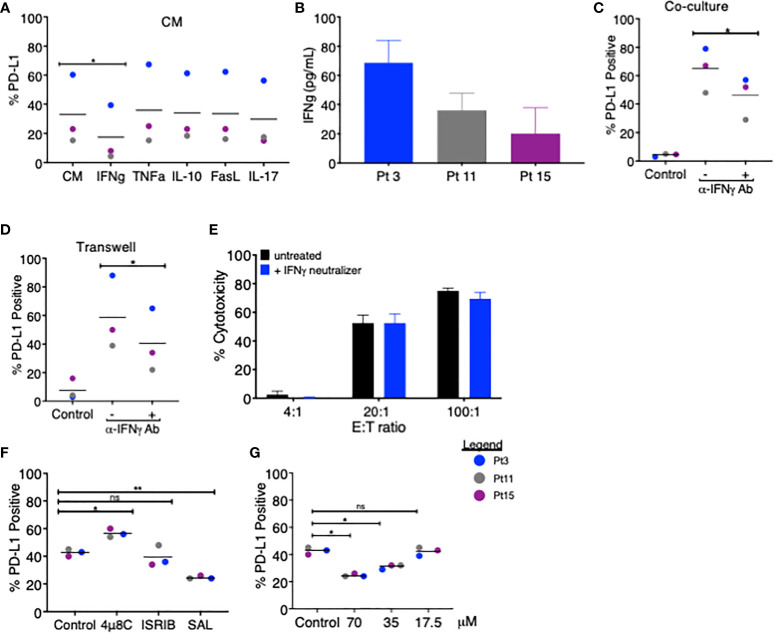
Blockade of IFNγ and PERK pathways partially attenuates PD-L1 upregulation on MDS cells. **(A)** MDS cells from three patients were analyzed for PD-L1 expression *via* flow cytometry after 18–20 h of treatment with coculture CM in the presence of blocking antibodies for IFNγ, TNFα, IL-10, FasL, or IL-17. **(B)** The amount of IFNγ in the CM of cocultures of MDS cells and autologous tumor-specific T cells from three patients was quantified using the QBeads supernatant assay. **(C)** MDS cells from three patients were analyzed for PD-L1 expression after an 18–20-h coculture **(C)** or in Transwell coculture **(D)** systems in the presence of an IFNγ neutralizer. **(E)** Neoantigen-specific T cells from Pt 3 were incubated overnight with autologous MDS cells at three effector:target (E:T) ratios with or without an IFNγ neutralizer. **(F)** MDS cells from three patients (Pt 3, 11, and 15) were analyzed for PD-L1 expression after an 18–20-h coculture with autologous tumor-specific T cells in the presence of 4µ8C (30 µM), ISRIB (1 µM), or Salubrinal (SAL, 70 µM). PD-L1 positivity was calculated by subtracting the percentage of PD-L1-positive cells at baseline from the percentage of PD-L1-positive cells after coculture. **(G)** MDS cells from three patients were analyzed for PD-L1 expression after 18–20 h of coculture with autologous tumor-specific T cells in the presence of 17.5, 35, and 70 µM Salubrinal. PD-L1 positivity was calculated as in panel **(A)** p values are the result of paired t-test, and bars represent the median. *p < 0.05, **p < 0.01. ns, not significant.

We next sought to determine the mechanistic basis for PD-L1 upregulation in MDS cells following treatment with coculture CM. Previous reports showed that PD-L1 can be induced in MDS cells by IFNγ and TNFα (9, 10), although a variety of other factors (i.e., IL-10, FasL, and IL-17A) have been shown to upregulate PD-L1 in other cancer types (11, 12). We treated MDS cells derived from three patients with autologous coculture CM in the presence of blocking antibodies for IFNγ, TNFα, IL-10, FasL, and IL-17 and compared the PD-L1 expression. Only the anti-IFNγ antibody reduced the PD-L1 expression on MDS cells, albeit by only 1.9-fold over coculture CM control (**Figure 2A**). In line with this finding, we detected IFNγ in all three coculture CM used in this experiment (range 20–68 pg/ml) (**Figure 2B**). We then added the anti-IFNγ antibody to the MDS:T cell coculture (**Figure 2C**) and Transwell (**Figure 2D**) systems and found a similar reduction in PD-L1 expression (1.4-fold and 1.5-fold, respectively). Together, these findings suggest that IFNγ is produced when T cells are cocultured with autologous MDS cells and that in turn IFNγ acts in a paracrine manner on MDS cells to upregulate PD-L1. However, blocking IFNγ had only a modest effect on PD-L1 upregulation and no effect on T cell-mediated lysis of MDS cells (**Figure 2E**), suggesting that PD-L1 expression in these experimental conditions may be the result of yet another mechanism acting in combination with IFNγ. To this end, we explored the contribution of the (UPR), an evolutionarily conserved homeostatic mechanism that cells use to cope with metabolic (e.g., hypoxia or nutrient starvation) or other cellular/environmental stressors (13). The UPR is an emerging regulator of the tumor:immune interface (14) since the induction of the UPR results in the upregulation of PD-L1 on macrophages in solid tumors (15). Here we performed MDS:T-cell coculture experiments in the presence of a panel of small-molecule inhibitors of UPR branches: (a) 4µ8C, an inhibitor of the inositol-requiring enzyme 1 (IRE1α); (b) Salubrinal, an inhibitor of eIF2α dephosphorylation downstream of PERK; and (c) ISRIB, a small molecule that rescues translation in the presence of eIF2α phosphorylation (16). We found that, of the inhibitors tested, only Salubrinal reduced PD-L1 upregulation on MDS cells (**Figures 2F**, **G**) suggesting that the PERK pathway, but not IRE1α, is involved in PD-L1 upregulation on MDS cells.

### PD-L1 expression does not affect the ability of TA-specific T cells to kill autologous MDS cells

**Figure 3:**
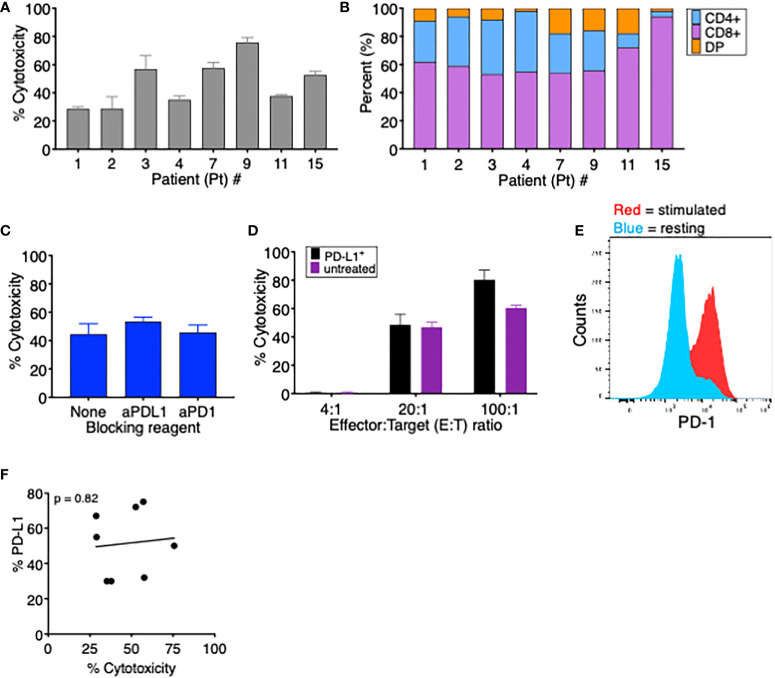
PD-L1 expression does not affect tumor-specific T cells’ ability to kill autologous MDS cells. **(A)** Percent cytotoxicity of MDS cells cultured with autologous tumor-specific T cells at an effector: target (E:T) ratio of 20:1. **(B)** Frequency of CD4^+^, CD8^+^, and CD4^+^CD8^+^ double-positive (DP) T cells among tumor-specific T cells for the eight patients analyzed in this study. **(C)** Percent cytotoxicity of tumor-specific T cells cultured with autologous MDS cells in the presence of the blocking reagents anti-PD-1 and anti-PD-L1. **(D)** Percent cytotoxicity of MDS cells from Pt 3 that were (PD-L1^+^, black bars) or were not (unstimulated, purple bars) induced to upregulate PD-L1 and then cultured for 18 h with autologous tumor-specific T cells. To induce PD-L1 upregulation, MDS cells were treated overnight (16–18 h) with 15 ng soluble IFNγ; 96% of MDS tumor cells were PD-L1^+^ prior to coculture. **(E)** Expression of PD-1 on resting (blue) or stimulated (red) T cells from Pt 3. **(F)** Correlation between percent PD-L1^+^ MDS cells after coculture CM treatment and percent cytotoxicity when cocultured with autologous tumor-specific T cells. p value was calculated by Spearman correlation. ^#^ number.

To assess the effect of T-cell-induced upregulation of PD-L1 on T-cell killing of MDS cells, we first measured the ability of TA-specific T cells to kill autologous MDS cells in a conventional cytotoxicity assay. In the eight MDS patients analyzed, we found that the percent cytotoxicity ranged from 28% to 76%, showing effective T-cell killing of all autologous patient-derived MDS cells (**Figure 3A**). When we analyzed the composition of TA-specific T cells in each patient, we found that the majority of T cells were CD8^+^ (range 53%–94%) (**Figure 3B**), with no significant correlation between percent cytotoxicity and the frequency of CD8^+^ T cells (data not shown). In addition, when Pt 5’s CD8^+^ and CD4^+^ cytotoxic T cells were isolated, both populations could induce PD-L1 expression (**Supplementary Figure 1A**) and lyse their autologous MDS targets at a similar frequency (**Supplementary Figure 1B**).

To evaluate if the PD-1/PD-L1 interaction interferes with lysis of target T cells, we performed cytotoxicity assays using TA-specific T cells and autologous MDS cells in the presence of either anti-PD-1 or anti-PD-L1 blocking antibodies. As seen in **Figure 3C**, the presence of blocking antibodies had no significant effect on T-cell-mediated cytotoxicity of MDS cells. We postulated that, since this patient’s MDS cells have low baseline PD-L1 expression (5% PDL1^+^), the cytotoxic cascade might occur more rapidly than the MDS cells increase PD-L1 expression. Therefore, we pretreated these MDS cells with or without soluble IFNγ to induce PD-L1 expression prior to the cytotoxicity assay. PD-L1 upregulation had no impact on the ability of T cells to kill MDS tumor cells since the percent lysis did not vary between PD-L1^+^ or PD-L1^-^ MDS cells at time 0 (**Figure 3D**) even though T cells expressed PD-1 (**Figure 3E**). In addition, in the eight cell lines tested in **Figure 3A**, there was no correlation between MDS cell PD-L1 expression and cytotoxicity by autologous TA-specific T cells (**Figure 3F**). This demonstrates that TA-specific T cells retained their ability to kill target cells expressing the inhibitory receptor PD-L1, implying either that the rapid process of killing is unaffected by PD-1:PD-L1 interaction or that the inhibitory signals may be overridden by alternative stimulatory co-receptors. In addition, we cannot exclude the possibility that repeated exposure could eventually lead to T-cell inhibition through the PD-1:PD-L1 axis.

## Discussion

Here we report that PD-L1 expression on MDS tumor cells is upregulated in the presence of autologous tumor-specific T cells. We determined that PD-L1 upregulation in this model system is mediated by a cell-contact-independent mechanism and involves secreted factors. PD-L1 upregulation occurred not only when MDS tumor cells and T cells were separated by a Transwell insert but also when MDS cells were exposed to the conditioned medium of MDS:T-cell cocultures. We also found that blocking either the PERK pathway or IFNγ inhibited PD-L1 upregulation and that PD-L1 expression on MDS cells did not inhibit their lysis by tumor-specific T cells.

Blocking IFNγ decreased PD-L1 upregulation on MDS tumor cells, which is consistent with previous findings on the role of IFNγ in inducing PD-L1 expression in a variety of different cell types (17, 18), including MDS cells (17). However, there is little evidence on which other signaling mechanisms may also contribute to PD-L1 induction on MDS cells. Here, we determined that beside IFNγ, the UPR may also play a role in increasing PD-L1 expression on MDS cells. We found that the PERK pathway of the UPR, but not IRE1α, contributes to T-cell-mediated PD-L1 induction in MDS. However, only Salubrinal, an inhibitor of eIF2α dephosphorylation, decreased PD-L1 surface expression. ISRIB, an inhibitor of eIF2A able to restore translation in the presence of eIF2α phosphorylation, did not decrease PD-L1 surface expression. This is surprising as they are both inhibitors of the PERK pathway, albeit with different mechanisms. A limitation of our study is that since these small-molecule inhibitors were added directly to the cocultures, their effects on MDS cells vs. T cells could not be distinguished. These results are at odds with reports that the IRE1α branch of the UPR regulates PD-L1 expression in tumor-associated macrophages (9) and in dendritic cells (19, 20). This argues that PD-L1 upregulation by the UPR is different in normal myeloid cells (IRE1α pathway) compared to MDS tumor cells (PERK pathway), an intriguing conundrum since MDS cells are tumor cells of myeloid origin.

A major objective of this study was to elucidate the potential reciprocal regulation between the PD-1:PD-L1 axis and adoptive cell therapy (ACT). Early results from clinical trials using ipilimumab after HMA failure show a low overall response rate of 3.4% (21), raising the possibility that the mechanism driving PD-L1 upregulation may matter—a consideration consistent with the fact that PD-L1 expression on antigen-presenting cells is more predictive of response to ICI than PD-L1 on tumor cells (22). In conclusion, we provide evidence for the upregulation of PD-L1 in MDS cells as a consequence of cognate and non-cognate interactions between MDS cells and autologous tumor-specific T cells, and that the *de novo* surface expression of PD-L1 on MDS cells is not sufficient on inhibit T-cell-mediated killing. These findings have direct relevance on the use of ICI in combination with ACT in MDS and suggest that blocking PD-1:PD-L1 would provide little or no benefit in a combination therapy. Given the efficacy of MDS cell lysis by neoantigen-specific T cells, our results suggest that ACT should be considered in combination with lymphodepletion and interleukin expansion strategies (5).

## Data availability statement

The raw data supporting the conclusions of this article will be made available by the authors, without undue reservation.

## Ethics statement

The studies involving human participants were reviewed and approved by University of California, San Diego Moores Cancer Center IRB NCT03072498. The patients/participants provided their written informed consent to participate in this study.

## Author contributions

VF and MZ designed the project. VF, AT, and HF performed all experiments and collected the data. TT was responsible for patient recruitment and critical review of the manuscript. VF, AT, SS, and MZ analyzed the data and wrote the manuscript. All authors contributed to the article and approved the submitted version.

## Acknowledgments

The authors thank Antonella Vitiello for her valuable input throughout the project and Raffaella and John Belanich for their support of this research.

## Conflict of interest

Authors VF, AT, and HF were employed by PersImmune, Inc., San Diego, CA, United States.

The remaining authors declare that the research was conducted in the absence of any commercial or financial relationships that could be construed as a potential conflict of interest.

## Publisher’s note

All claims expressed in this article are solely those of the authors and do not necessarily represent those of their affiliated organizations, or those of the publisher, the editors and the reviewers. Any product that may be evaluated in this article, or claim that may be made by its manufacturer, is not guaranteed or endorsed by the publisher.

## Supplementary material

Supplementary Figure 1 CD8^+^ and CD4^+^ T cells contribute to upregulation of PD-L1 and lysis of MDS cells. **(A)** Percent cytotoxicity of tumor-specific CD8^+^, CD4^+^, and unseparated T cells incubated overnight with autologous MDS cells. **(B)** Percent PD-L1^+^ MDS cells after overnight incubation with autologous tumor-specific T cells.

## References

[1] Prebet T Gore SD Esterni B Gardin C Itzykson R Thepot S. Outcome of high-risk myelodysplastic syndrome after azacitidine treatment failure. J Clin Oncol (2011) 29(24):3322–7. doi: 10.1200/JCO.2011.35.8135 PMC4859209 21788559

[2] Fenaux P Mufti GJ Hellstrom-Lindberg E Santini V Finelli C Giagounidis A. Efficacy of azacitidine compared with that of conventional care regimens in the treatment of higher-risk myelodysplastic syndromes: A randomised, open-label, phase III study. Lancet Oncol (2009) 10(3):223–32. doi: 10.1016/S1470-2045(09)70003-8 PMC4086808 19230772

[3] Lubbert M Suciu S Baila L Rüter BH Platzbecker U Giagounidis A. Low-dose decitabine versus best supportive care in elderly patients with intermediate- or high-risk myelodysplastic syndrome (MDS) ineligible for intensive chemotherapy: Final results of the randomized phase III study of the European organisation for research and treatment of cancer leukemia group and the German MDS study group. J Clin Oncol (2011) 29(15):1987–96. doi: 10.1200/JCO.2010.30.9245 21483003

[4] Ferrari V Tarke A Fields H Ferrari L Conley T Ferrari F. *In vitro* induction of neoantigen-specific T cells in myelodysplastic syndrome, a disease with low mutational burden. Cytother (2021) 23(4):320–8. doi: 10.1016/j.jcyt.2020.10.003 33262074

[5] Tanaka TN Ferrari V Tarke A Fields H Ferrari L Ferrari F. Adoptive transfer of neoantigen-specific T-cell therapy is feasible in older patients with higher-risk myelodysplastic syndrome. Cytother (2021) 23(3):236–41. doi: 10.1016/j.jcyt.2020.11.003 33279399

[6] Wang X Bao Z Zhang X Li F Lai T Cao C. Effectiveness and safety of PD-1/PD-L1 inhibitors in the treatment of solid tumors: A systematic review and meta-analysis. Oncotar (2017) 8(35):59901–14. doi: 10.18632/oncotarget.18316 PMC5601788 28938692

[7] Yang H Bueso-Ramos C DiNardo C Estecio MR Davanlou M Geng QR. Expression of PD-L1, PD-L2, PD-1 and CTLA4 in myelodysplastic syndromes is enhanced by treatment with hypomethylating agents. Leukemia. (2014) 28(6):1280–8. doi: 10.1038/leu.2013.355 PMC4032802 24270737

[8] Montalban-Bravo G Garcia-Manero G. Myelodysplastic syndromes: 2018 update on diagnosis, risk-stratification and management. Am J Hematol (2018) 93(1):129–47. doi: 10.1002/ajh.24930 29214694

[9] Garcia-Diaz A Shin DS Moreno BH Saco J Escuin-Ordinas H Rodriguez GA. Interferon receptor signaling pathways regulating PD-L1 and PD-L2 expression. Cell Rep (2017) 19(6):1189–201. doi: 10.1016/j.celrep.2017.04.031 PMC6420824 28494868

[10] Wang X Yang L Huang F. Inflammatory cytokines IL-17 and TNF-alpha up-regulate PD-L1 expression in human prostate and colon cancer cells. Immunol Lett (2017) 184:7–14. doi: 10.1016/j.imlet.2017.02.006 28223102 PMC5362328

[11] Qian Q Wu C Chen J Wang W. Relationship between IL10 and PD-L1 in liver hepatocellular carcinoma tissue and cell lines. BioMed Res Int (2020) 2020:8910183. doi: 10.1155/2020/8910183 32724815 PMC7381951

[12] Gu YZ Xue Q Chen YJ Yu G Qing M Shen Y. Different roles of PD-L1 and FasL in immunomodulation mediated by human placenta-derived mesenchymal stem cells. Hum Immunol (2013) 74(3):267–76. doi: 10.1016/j.humimm.2012.12.011 23261407

[13] Walter P Ron D. The unfolded protein response: From stress pathway to homeostatic regulation. Science. (2011) 334(6059):1081–6. doi: 10.1126/science.1209038 22116877

[14] Rodvold JJ Mahadevan NR Zanetti M. Immune modulation by ER stress and inflammation in the tumor microenvironment. Cancer Lett (2016) 380(1):227–36. doi: 10.1016/j.canlet.2015.09.009 26525580

[15] Batista A Rodvold JJ Xian S Searles SC Lew A Iwawaki T. IRE1alpha regulates macrophage polarization, PD-L1 expression, and tumor survival. PloS Biol (2020) 18(6):e3000687. doi: 10.1371/journal.pbio.3000687 32520957 PMC7307794

[16] Gonzalez-Teuber V Albert-Gasco H Auyeung VC Papa FR Mallucci GR Hetz C. Small molecules to improve ER proteostasis in disease. Trends Pharmacol Sci (2019) 40(9):684–95. doi: 10.1016/j.tips.2019.07.003 31377018

[17] Chen S Crabill GA Pritchard TS McMiller TL Wei P Pardoll DM. Mechanisms regulating PD-L1 expression on tumor and immune cells. J immunother cancer. (2019) 7(1):305. doi: 10.1186/s40425-019-0770-2 31730010 PMC6858680

[18] Mimura K Teh JL Okayama H Shiraishi K Kua L Koh V. PD-L1 expression is mainly regulated by interferon gamma associated with JAK-STAT pathway in gastric cancer. Cancer Sci (2018) 109(1):43–53. doi: 10.1111/cas.13424 29034543 PMC5765310

[19] Cubillos-Ruiz JR Silberman PC Rutkowski MR Chopra S Perales-Puchalt A Song M. ER stress sensor XBP1 controls anti-tumor immunity by disrupting dendritic cell homeostasis. Cell. (2015) 161(7):1527–38. doi: 10.1016/j.cell.2015.05.025 PMC4580135 26073941

[20] Cubillos-Ruiz JR Bettigole SE Glimcher LH. Molecular pathways: Immunosuppressive roles of IRE1alpha-XBP1 signaling in dendritic cells of the tumor microenvironment. Clin Cancer Res (2016) 22(9):2121–6. doi: 10.1158/1078-0432.CCR-15-1570 PMC4854763 26979393

[21] Zeidan AM Knaus HA Robinson TM Towlerton AMH Warren EH Zeidner JF. A multi-center phase I trial of ipilimumab in patients with myelodysplastic syndromes following hypomethylating agent failure. Clin Cancer Res (2018) 24(15):3519–27. doi: 10.1158/1078-0432.CCR-17-3763 PMC6680246 29716921

[22] Lin H Wei S Hurt EM Green MD Zhao L Vatan L. Host expression of PD-L1 determines efficacy of PD-L1 pathway blockade-mediated tumor regression. J Clin Invest. (2018) 128(2):805–15. doi: 10.1172/JCI96113 PMC5785251 29337305

