# Supplementary figures and images for: Tumor-specific T cell-mediated upregulation of PD-L1 in myelodysplastic syndrome cells does not affect T-cell killing
Source: Front Oncol. 2022 Aug 5;12:915629. doi: 10.3389/fonc.2022.915629 (PMC9389224; doi:10.3389/fonc.2022.915629)

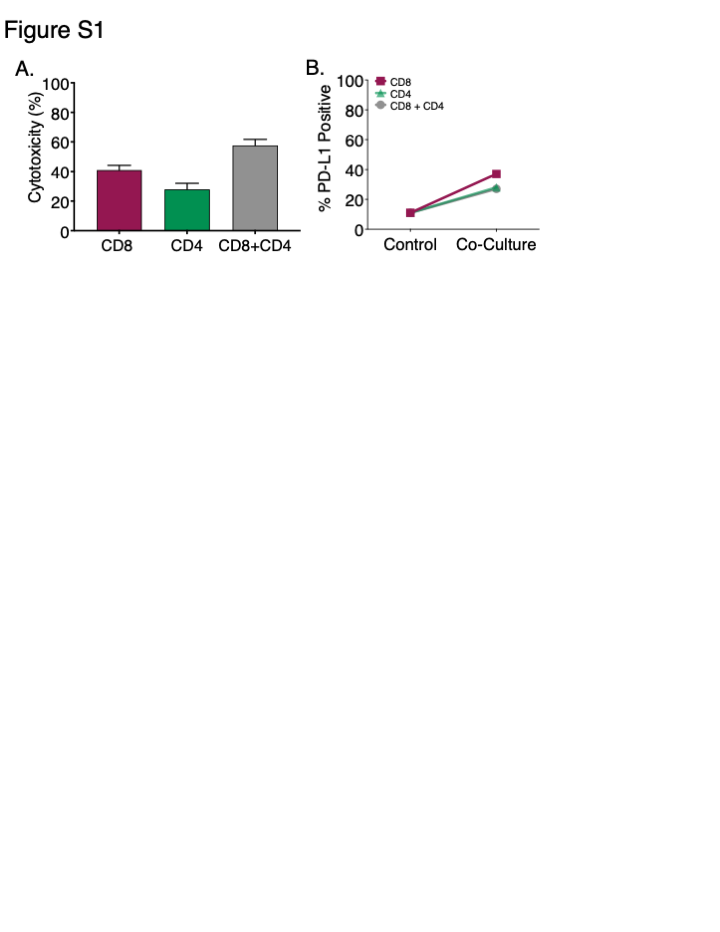

Supplement: Supplementary Figure 1 — CD8+ and CD4+ T cells contribute to upregulation of PD-L1 and lysis of MDS cells. (A) Percent cytotoxicity of tumor-specific CD8+, CD4+, and unseparated T cells incubated overnight with autologous MDS cells. (B) Percent PD-L1+ MDS cells after overnight incubation with autologous tumor-specific T cells. [file Image_1.tiff]
